# Craving as a transdiagnostic marker of addiction? A perspective for behavioral addictions

**DOI:** 10.3389/fnbeh.2026.1770895

**Published:** 2026-02-24

**Authors:** Axel Allache, Marc Auriacombe, Jean-Marc Alexandre, Fuschia Serre

**Affiliations:** 1 University of Bordeaux, Bordeaux, France; 2 CNRS, SANPSY, UMR 6033, Bordeaux, France; 3 Pôle Inter-établissement d’Addictologie, CH Ch. Perrens and CHU de Bordeaux, Bordeaux, France

**Keywords:** behavioral addictions, craving, diagnosis, DSM, mental health, transdiagnostic

## Abstract

The addiction term is sometimes overused, both in the media and popular discourse, to describe excessive engagement in everyday behaviors such as sugar consumption, screen use, or physical exercise. This overuse may reflect the fact that rewarding behaviors, much like the use of rewarding substances, can lead to repeated use, occasionally beyond what is considered reasonable and with significant negative consequences. This raises fundamental questions: by which criteria can an addiction be identified? Is the phenomenon the same for substances and behaviors? This perspective article proposes to explore these questions by examining the relevance of craving, defined as an intense, persistent, but involuntary desire to use a specific substance/behavior. Although craving is well-established as a core criterion for substance addictions, with strong prognostic value, clinical utility, it has not yet been formally integrated into diagnostic classifications for behavioral addictions. This perspective article reviews recent evidence supporting that craving may represent a transdiagnostic construct across substance and behavioral addictions.

## Introduction

Substance use disorder (SUD), or addiction, is a psychiatric disorder characterized by the loss of control over substance use, leading to persistent use despite related negative consequences and a prioritization of substance use over alternative rewards and meaningful activities (American Psychiatric Association, 2013; Auriacombe et al., 2018). In diagnostic classifications, the extension of the concept of addiction to rewarding behaviors void of substance intake has progressively evolved. In the DSM-IV, pathological gambling was listed among “Impulse-Control Disorders.” In 2013, the DSM-5 opened the “Substance-Related and Addictive Disorders” category to both substance and behavioral addictions, including pathological gambling (Petry et al., 2013, 2014), whereas Internet Gaming Disorder (IGD) was included as a disorder for further study (American Psychiatric Association, 2013). In parallel, the ICD-11 officially recognized in 2018 gambling disorder and gaming disorder as addictions (World Health Organization, 2018). At the same time, the scientific literature accumulates behavioral, psychological and neurobiological data suggesting similarities between substance and behavioral addictions (Potenza, 2009; Kim and Hodgins, 2018).

In the media and in popular discourse, addiction also became a term, sometimes overused, to describe excessive engagement in everyday behaviors such as sugar consumption, screen use, or physical exercise. This overuse may reflect the fact that rewarding behaviors, much like the use of rewarding substances, can lead to repeated use, occasionally beyond what is considered reasonable, and sometimes difficult to control. This raises important questions: by which criteria can an addiction be identified? Is the phenomenon the same for substances and behaviors?

## The question of the definition of behavioral addictions

The inclusion of some behaviors in diagnostic classifications of addiction has extended the scientific interest toward other everyday rewarding activities that may become excessive and cause distress or impairment. This trend has also been amplified by the rise of the internet, which facilitates access to some of these behaviors (Young, 1998; Kuss and Griffiths, 2012). Consequently, a wide range of rewarding behaviors or activities, which can sometimes lead to excessive use, both online (e.g., compulsive buying, social media use, cyberchondria, or pornography use/compulsive sexual behavior), or offline (e.g., excessive exercise, work, or eating), have been proposed as potentially at risk for developing a behavioral addiction (Demetrovics and Griffiths, 2012; Brand et al., 2025). However, some authors warn against over-pathologization of common behaviors, and the risk of blurring the boundaries between “normal” and “pathological” behaviors, emphasizing that the frequency or amount of use is not sufficient to qualify for an addiction (Billieux et al., 2015). For example, excessive smartphone use or binge-watching remains debated as to whether they constitute true addictions (Panova and Carbonell, 2018; Ort et al., 2021). These examples highlight the urgent need to clarify the boundary between intensive, non-pathological engagement and a genuine pathology such as addiction.

To conceptualize and diagnose a potential behavioral addiction, several methods have been suggested. One method consists of exploring if the behavior fits into the construct of substance addiction, and transposes the SUD diagnostic criteria to this behavior (Grant et al., 2010). This “transdiagnostic approach” could be supported by overlapping symptoms and mechanisms observed between behavioral and substance addictions. An alternative approach is to examine this behavior as a new and distinct phenomenon, rather than as part of the addiction construct. This “behavior-specific approach” advocates the development of a behavior-specific diagnostic criteria set, grounded in a multidimensional understanding of the disorder, and validated through longitudinal data, psychometric analyses, and empirical research (Billieux et al., 2015, 2017; Castro-Calvo et al., 2021; Baggio et al., 2022). These two approaches have their advantages. The transdiagnostic approach could facilitate research and clinical recognition of behavioral addictions. This would provide a unified conceptual framework for all addictive behaviors, while building on established diagnostic structures and validated assessment tools. It would also facilitate the comparison of different addictions, accelerating the identification of common mechanisms and capturing the potential transdiagnostic core of addiction (Shaffer et al., 2004; Kim and Hodgins, 2018; Garrison et al., 2023). Conversely, the behavior-specific approach may help avoid over-pathologization by reducing the risk of false-positive diagnoses that could result from applying irrelevant criteria, while instead incorporating criteria that may be more specifically relevant to certain behavioral addictions (Billieux et al., 2015; Starcevic, 2016; Starcevic and Billieux, 2017; Castro-Calvo et al., 2021; Fineberg et al., 2022; Flayelle et al., 2025).

This debate over whether diagnostic criteria should be standardized across all addictions is far from new. Throughout the revision process related to the DSM-5, the transdiagnostic approach has been favored for the different substances, i.e., applying the same set of criteria to all SUD. This decision was grounded in the many common biological mechanisms, clinical symptoms and treatment responses across SUD, albeit the fact that not all individual criteria have the same psychometric characteristics when comparing between substances (Hasin et al., 2013; Koob and Volkow, 2016; Kervran et al., 2020; Volkow and Blanco, 2023).

We propose to reconcile these apparently divergent approaches (behavior-specific versus transdiagnostic) to conceptualize and diagnose behavioral addictions. We argue that such reconciliation may lie in a deeper understanding of the core impairment underlying addiction, whether substance-related or behavioral. Both the DSM-5 and ICD-11 emphasize that addictive use must be identified as problematic, i.e., causing significant distress and functional impairment, and rely on a concise set of central criteria regarded as the “core” features of addiction, primarily related to loss of control (Potenza, 2014; Koob and Volkow, 2016). In our opinion, the craving criterion may represent a promising avenue for capturing this loss of control from a transdiagnostic perspective.

## Craving as a central marker in substance use disorder

Even though its definition remains debated, craving is generally described, in the field of addiction, as an intense desire or urge to use a substance (Sayette et al., 2000; Cherpitel et al., 2010; American Psychiatric Association, 2013), which persists beyond the withdrawal period, and that can be distinguished from a “normal” desire by its involuntary and unwanted nature (Auriacombe et al., 2018; Vafaie and Kober, 2022).

To avoid confusion, it is important to note that in English, craving is a term that is neither specific to addiction nor restricted to substance use. As such, craving is not pathological by nature, and could describe normal motivational and adaptive mechanisms that can emerge in response to any deprivation, such as food craving during fasting or craving for social contact during periods of isolation (Tomova et al., 2020). Adaptive and pathological craving are based on largely shared motivational circuits, and pathological craving in addiction is distinguished not by the nature of the mechanisms involved, but by its dysregulation, persistence beyond deprivation, and dominance over alternative rewards (Robinson and Berridge, 2008; Koob and Volkow, 2010; Volkow et al., 2016). The term craving is also used to describe compulsive urges (for food or particular eating behaviors) in eating disorders [for review (Cornil et al., 2026)], which interestingly have been frequently compared to addiction, and may partially overlap with food addiction (Meule and Kübler, 2012; Fauconnier et al., 2020; Mallorquí-Bagué et al., 2020; Chapron et al., 2023; Collombat et al., 2024). In addition, craving is also employed to describe anxiety-driven urges aimed at alleviating negative emotional states in obsessive–compulsive disorder (el-Guebaly et al., 2012; Jones and Bhattacharya, 2012). These forms of craving appear to differ in their underlying neurobiological substrates as well as in their responses to pharmacological treatment (el-Guebaly et al., 2012; Piquet-Pessoa and Fontenelle, 2016; Ferreira et al., 2021).

Accordingly, craving in its broad sense is not sufficient to distinguish addiction-related mechanisms from other motivational processes. Within the context of addiction, craving is more precisely conceptualized as a pathological motivational desire directed toward a particular rewarding object, characterized by its persistence independent of deprivation, its prioritization over alternative rewards, and its involuntary and unwanted nature (Robinson and Berridge, 2008; Koob and Volkow, 2010; Volkow et al., 2016; Auriacombe et al., 2018; Vafaie and Kober, 2022). In the remainder of this manuscript, the term craving refers to this definition of pathological craving in addiction.

At the diagnostic level, craving represents a core criterion across substance addictions. The introduction of craving as a diagnostic criterion for SUD in DSM-5, based on the growing body of evidence showing its major role in addiction, has sparked increased scientific interest (Sayette, 2016). Psychometric studies, such as Item Response Theory (IRT) analyses, examine how each criterion of a scale relates to (and is informative about) an underlying latent construct (Embretson and Reise, 2000; Sahin and Anil, 2017; Arend and Schäfer, 2019). IRT analyses have shown that craving was the least severe and most discriminant among the 11 DSM-5 SUD criteria when comparing across substances (Kervran et al., 2020) and samples of users (Chung et al., 2012; Gilder et al., 2014; Castaldelli-Maia et al., 2018; Kervran et al., 2021; Shmulewitz et al., 2023), suggesting that craving could represent an early manifestation of addictive processes, and could also be a sensitive marker of a transition from controlled to addictive use. More recently, some authors proposed that diagnostic criteria could be dynamically and causally mutually dependent, and thereby propose representation of a disorder as a network, i.e., a web of mutually influencing symptoms (Borsboom and Cramer, 2013), allowing exploration of their interrelationships (Fried, 2015; Epskamp et al., 2017; Schlechter et al., 2021). Such network analyses offer a window for formulating hypotheses about how disorders develop, are maintained and could be treated. Especially, centrality inferences can be understood to reflect how connected and thus potentially clinically relevant a diagnostic criterion is in a network. Such analyses applied to SUD criteria revealed that craving was a symptom that consistently remained in terms of centrality regardless of the substance explored, confirming its central place in the diagnosis of addiction (Gauld et al., 2023; Shmulewitz et al., 2025). These elements, observed across substances, support the hypothesis that craving is a transdiagnostic marker applicable to any substance addiction.

Interestingly, beyond its diagnostic value, craving is by nature a dynamic phenomenon whose frequency and intensity vary over time depending on internal and external factors (Sayette et al., 2000; Enkema et al., 2020). The use of Ecological Momentary Assessment (EMA), a method collecting information in natural contexts through repeated questionnaires completed directly on smartphone applications (Stone and Shiffman, 1994), has allowed to capture craving fluctuations in daily life (Serre et al., 2012, 2015, 2025). As a state, craving has shown prognostic relevance, as fluctuations in its intensity can predict subsequent substance use within a few hours, but also the risk of relapse over the course of several years (Fatseas et al., 2015; Serre et al., 2015, 2025; Auriacombe et al., 2018; Vafaie and Kober, 2022; Baillet et al., 2024a), documented for many different substance addictions, thereby confirming craving as a key target for intervention. However, craving does not always predict persistence of use, and its predictive value for relapse depends on both the timing and methodology of its assessment (Serre et al., 2015; Vafaie and Kober, 2022), as well as on its interaction with other psychological and contextual factors (Serre et al., 2025).

## Craving in behavioral addiction

The craving phenomenon is also reported for behavioral addictions, including gambling disorder (Antons et al., 2023; Mallorqui-Bague et al., 2023; Westerberg et al., 2025), gaming disorder (Zhou et al., 2022; Park et al., 2023), problematic pornography use/compulsive sexual behavior (Antons et al., 2020; Antons and Matthias, 2020; Miele et al., 2023), food-related disorders (Roefs et al., 2019; Chapron et al., 2023), problematic social media use/problematic smartphone use (De-Sola et al., 2017; Moretta and Buodo, 2018) and excessive exercise (Heirene et al., 2016). Many authors highlight this phenomenon as relevant across behavioral addictions (Kim and Hodgins, 2018; Brand et al., 2019; Castro-Calvo et al., 2021). Similarly, despite the challenge of modeling craving in animals (Field and Kersbergen, 2020), some animal models of behavioral and non-substance addictions have reported craving-like responses to natural rewards such as sucrose or gaming (Grimm et al., 2005; Casile et al., 2025), suggesting that craving is neither intrinsically drug-specific nor necessarily dependent on direct external pharmacological effects or fundamental physiological needs. These models therefore offer the potential for an interesting framework to disentangle the components of craving that reflect adaptive motivational processes from those that index maladaptive, addiction-like mechanisms. However, despite being included in some assessment or screening tools for behavioral addictions (De-Sola et al., 2017; Panova and Carbonell, 2018; Fineberg et al., 2022; Chapron et al., 2023; Miele et al., 2023; Park et al., 2023; Antons et al., 2025), to date, craving has not been included as a diagnostic criterion for behavioral addictions in DSM-5/ICD-11 classifications.

At the neurobiological level, craving for different addictive behaviors has been associated with activation in rewards-related cerebral networks, particularly the ventral striatum and orbitofrontal regions involved in assigning value to stimuli. These motivational states also mobilize salience and emotional regulation circuits, such as the insula and anterior cingulate cortex, as well as prefrontal regions responsible for executive control (Potenza, 2008; Zhang et al., 2021; Park et al., 2023). Interestingly, these circuits show a large overlap with those involved in SUD.

At the behavioral level, craving for gaming, gambling, and social networks can be induced by cue exposure (Ashrafioun et al., 2012; Shin et al., 2018; Ciccarelli et al., 2019; Zhou et al., 2022), and craving intensity has been associated with severity and risk of relapse in gaming and gambling disorders (Quintero et al., 2020; Mallorqui-Bague et al., 2023; Westerberg et al., 2025). In daily life, EMA studies demonstrated that an increase in craving was associated with an increased probability of gambling (Dowling et al., 2021; Hawker et al., 2021) and pornography use (Henry et al., 2024) in the following hours, as it has been previously highlighted across substance addictions (Serre et al., 2015, 2018, 2025).

## Perspective: craving as a transdiagnostic marker across all addiction types, substances and behaviors?

The findings presented above suggest that craving, well-characterized in SUD regarding neurobiological pathways and behavioral consequences, also constitutes an important feature of behavioral addictions. This leads us to propose that craving may represent a transdiagnostic cornerstone for a shared framework encompassing both substances and behavioral addictions, of value for both mechanistic understanding, diagnosis and treatment.

Several findings support the hypothesis that craving is a central characteristic across different forms of addiction. Psychometric analyses of a craving scale, applied to both substance and behavioral addictions, have identified craving as a latent factor shared across several addictions (Antons et al., 2025). A systematic review of craving assessment tools across substances and behaviors also supported the conceptualization of craving as a transdiagnostic phenomenon (Miele et al., 2023). At the neurobiological level, studies revealed the involvement of similar neural mechanisms and networks in predicting craving across different addictions (Antons et al., 2024). Converging evidence also emerged from deep transcranial magnetic stimulation studies, with reductions in craving obtained thanks to similar stimulation target patterns across both substance and behavioral addictions (Del Mauro et al., 2025). Nonetheless, further studies are needed.

At the diagnosis level, a first step could be to assess the diagnostic value of the craving criterion within the context of behavioral addictions, as it has been previously done to define the set of criteria for SUD in the DSM-5. For this purpose, a set of criteria, including both SUD-derived criteria and other potentially relevant ones specific to each addictive behavior (i.e., derived from qualitative interviews) could be assessed in a large sample of people who regularly engage in the behavior in question, for psychometric analyses. In the context of natural behaviors, because craving may overlap with adaptive motivation, its definition should be explicitly specified at the time of measurement. In SUD, IRT analyses have consistently demonstrated a unifactorial dimension and identified craving as one of the most discriminating and least difficult criteria to meet (Kervran et al., 2020). Similarly, when the DSM-5 criteria were adapted to food, unidimensionality was also confirmed and craving showed good discriminative value (Chapron et al., 2023). Drawing on these results, we hypothesize that the craving criterion will also appear to be one of the most psychometrically relevant criteria in behavioral addictions. In the same way, network analyses have highlighted the centrality of craving within the criteria networks of several SUDs (alcohol, cannabis, tobacco, opioids, and cocaine) (Gauld et al., 2023; Shmulewitz et al., 2025). Extending network analyses to behavioral addictions would allow to examine how craving is central within these disorders.

Beyond its value and utility for establishing diagnosis, recognizing the relevance of craving for behavioral addictions will also rely on demonstrating its etiological and predictive role in behavioral addictions. An important further step will be to explore how craving fluctuations relate to engagement in addictive behaviors and relapse. As discussed previously, several studies have begun to link craving intensity to addictive behavior practice, based on both laboratory and daily life data (Dowling et al., 2021; Hawker et al., 2021; Zhang et al., 2021; Ciccarelli et al., 2022; Zhou et al., 2022; Henry et al., 2024). The use of new approaches combining EMA and dynamic network analyses would make it possible to assess, at the within-person level, how craving interacts with other components (emotional, contextual, or cognitive components) that may influence behavior or relapse. In substance addiction, dynamic network analyses have shown that higher craving, combined with lower self-efficacy, are the best predictors of substance use in the following hours (Serre et al., 2025). We hypothesize that, in behavioral addictions, craving may retain this central role. Comparing substance and behavioral addictions networks could also help reveal factors that exert similar, or, conversely, distinct, influences, thereby contributing to a clearer identification of the central and common core that may support the development of a unified framework for all addictions.

Another important step to demonstrate the transdiagnostic value of craving across substance and behavioral addictions will be to explore if it shares biological substrates in both SUD and behavioral addictions. Some studies have already highlighted similarities in the neurobiology associated with craving (Antons et al., 2024; Del Mauro et al., 2025). In SUD, several studies have shown that craving was also associated to physiological changes in the autonomic nervous system (heart rate, electrodermal activity), suggesting the existence of a physiological signature specific to craving (Kennedy et al., 2015; Carreiro et al., 2020; Baillet et al., 2024b). These results support the idea of a biological marker for craving, measurable in daily life, but it remains necessary to extend these investigations to behavioral addictions to determine whether a comparable signature can be observed.

## Conclusion

This paper has examined contemporary issues related to behavioral addictions, emphasizing the central and transdiagnostic role of craving.

### By what criteria can an addiction be identified?

A consensus emerged across the different classifications to restrict the term addiction to substance/behavior use identified as problematic, i.e., causing significant distress and functional impairment. Authors from the field emphasized the importance of not pathologizing behaviors on intensity/frequency criteria (Billieux et al., 2015; Castro-Calvo et al., 2021; Flayelle et al., 2025). For this, identification of what is really central and impaired in addiction, and proposing it as a necessary prerequisite for diagnosis, may be an important step. A growing body of research tends to demonstrate the role played by craving as a driver of addictive behavior. However, more data are needed to demonstrate its central role in the loss of control in addictive behaviors. We suggest that systematically adding a craving item to behavioral addictions assessment tools is necessary to broaden our understanding of its mechanistic role. Subsequent psychometric studies could examine how informative this craving criterion is in relation to behavioral addiction, as well as its discriminative power in identifying individuals with the disorder.

### Are behavioral addictions genuinely the same phenomenon as substance-related addiction?

The integration of substance and behavioral addictions into a single diagnostic entity in DSM-5 and ICD-11 already indicates an acknowledgment that addiction cannot be thought solely in terms of the substance consumed or behavior practiced, but be better captured by its underlying processes and loss of control mechanisms. The literature already highlights numerous similarities, both at the behavioral and neurobiological levels. Continuing this line of research appears essential to further consolidate these findings. At the same time, the question of whether differences exist beyond these similarities seems to hinder the merging of substance and behavioral addictions. From our perspective, such differences do indeed exist, even among substances themselves, but they are more often related to the consequences of use or to causal factors. Refocusing on the mechanistic core of the addiction pathology could allow us to move beyond these differences, and we believe that craving is one such core feature.

## Data Availability

The original contributions presented in this study are included in this article/supplementary material, further inquiries can be directed to the corresponding author.

## References

[B1] American Psychiatric Association (2013). *Diagnostic and Statistical Manual of Mental Disorders.* Washington, DC: American Psychiatric Association.

[B2] Antons S. Brand M. Potenza M. N. (2020). Neurobiology of cue-reactivity, craving, and inhibitory control in non-substance addictive behaviors. *J. Neurol. Sci.* 415:116952. 10.1016/j.jns.2020.116952 32534370

[B3] Antons S. Brandtner A. Oelker A. Trotzke P. Wegmann E. Brand M. (2025). Psychometric evaluation of a trans-addiction craving questionnaire: The Craving Assessment Scale for Behavioral Addictions and Substance-use Disorders (CASBAS). *Addiction* 121 400–412. 10.1111/add.70197 41013925 PMC12779585

[B4] Antons S. Matthias B. (2020). Inhibitory control and problematic Internet-pornography use - The important balancing role of the insula. *J. Behav. Addict.* 9 58–70. 10.1556/2006.2020.00010 32359231 PMC8935194

[B5] Antons S. Yip S. W. Lacadie C. M. Dadashkarimi J. Scheinost D. Brand M. (2023). Connectome-based prediction of craving in gambling disorder and cocaine use disorder. *Dial. Clin. Neurosci.* 25 33–42. 10.1080/19585969.2023.2208586 37190759 PMC10190201

[B6] Antons S. Yip S. W. Lacadie C. M. Dadashkarimi J. Scheinost D. Brand M. (2024). Prediction of craving across studies: A commentary on conceptual and methodological considerations when using data-driven methods. *J. Behav. Addict.* 13 695–701. 10.1556/2006.2024.00050 39356557 PMC11457034

[B7] Arend M. G. Schäfer T. (2019). Statistical power in two-level models: A tutorial based on Monte Carlo simulation. *Psychol. Methods* 24 1–19. 10.1037/met0000195 30265048

[B8] Ashrafioun L. McCarthy A. Rosenberg H. (2012). Assessing the impact of cue exposure on craving to gamble in university students. *J. Gambl. Stud.* 28 363–375. 10.1007/s10899-011-9262-0 21853232

[B9] Auriacombe M. Serre F. Denis C. Fatséas M. (2018). “Diagnosis of addictions,” in *The Routledge Handbook of Philosophy and Science of Addiction*, 1 Edn, eds Pickard H. Ahmed S. H. (New York, NY: Routledge).

[B10] Baggio S. Starcevic V. Billieux J. King D. L. Gainsbury S. M. Eslick G. D. (2022). Testing the spectrum hypothesis of problematic online behaviors: A network analysis approach. *Addict. Behav.* 135:107451. 10.1016/j.addbeh.2022.107451 35939963

[B11] Baillet E. Auriacombe M. Romao C. Garnier H. Gauld C. Vacher C. (2024a). Craving changes in first 14 days of addiction treatment: An outcome predictor of 5 years substance use status? *Transl. Psychiatry* 14:497. 10.1038/s41398-024-03193-3 39695105 PMC11655831

[B12] Baillet E. Serre F. Si-Mohammed H. Romao C. Bruneau A. Vacher C. (2024b). The autonomic craving signature: Physiological signals as a daily-life biomarker of craving. *Res. Sq.* 10.21203/rs.3.rs-4432651/v1 [Preprint].

[B13] Billieux J. King D. L. Higuchi S. Achab S. Bowden-Jones H. Hao W. (2017). Functional impairment matters in the screening and diagnosis of gaming disorder. *J. Behav. Addict.* 6 285–289. 10.1556/2006.6.2017.036 28816514 PMC5700712

[B14] Billieux J. Schimmenti A. Khazaal Y. Maurage P. Heeren A. (2015). Are we overpathologizing everyday life? A tenable blueprint for behavioral addiction research. *J. Behav. Addict.* 4 119–123. 10.1556/2006.4.2015.009 26014667 PMC4627665

[B15] Borsboom D. Cramer A. O. J. (2013). Network analysis: An integrative approach to the structure of psychopathology. *Ann. Rev. Clin. Psychol.* 9 91–121. 10.1146/annurev-clinpsy-050212-185608 23537483

[B16] Brand M. Antons S. Bothe B. Demetrovics Z. Fineberg N. A. Jimenez-Murcia S. (2025). Current advances in behavioral addictions: From fundamental research to clinical practice. *Am. J. Psychiatry* 182 155–163. 10.1176/appi.ajp.20240092 39659159

[B17] Brand M. Wegmann E. Stark R. Müller A. Wölfling K. Robbins T. W. (2019). The Interaction of Person-Affect-Cognition-Execution (I-PACE) model for addictive behaviors: Update, generalization to addictive behaviors beyond internet-use disorders, and specification of the process character of addictive behaviors. *Neurosci. Biobehav. Rev.* 104 1–10. 10.1016/j.neubiorev.2019.06.032 31247240

[B18] Carreiro S. Chintha K. K. Shrestha S. Chapman B. Smelson D. Indic P. (2020). Wearable sensor-based detection of stress and craving in patients during treatment for substance use disorder: A mixed methods pilot study. *Drug Alcohol Dependence* 209:107929. 10.1016/j.drugalcdep.2020.107929 32193048 PMC7197459

[B19] Casile A. Marraudino M. Bonaldo B. Micioni, Di Bonaventura M. V. Nasini S. (2025). Novel rat model of gaming disorder: Assessment of social reward and sex differences in behavior and c-Fos brain activity. *Psychopharmacology* 242 1103–1122. 10.1007/s00213-024-06576-y 38575792 PMC12043766

[B20] Castaldelli-Maia J. M. Andrade L. H. Storr C. L. Viana M. C. Andrade A. G. Martins S. S. (2018). The latent trait of ICD-11 nicotine dependence criteria: Dimensional and categorical phenotypes. *Psychiatry Res.* 266 275–283. 10.1016/j.psychres.2018.03.018 29605101

[B21] Castro-Calvo J. King D. L. Stein D. J. Brand M. Carmi L. Chamberlain S. R. (2021). Expert appraisal of criteria for assessing gaming disorder: An international Delphi study. *Addiction* 116 2463–2475. 10.1111/add.15411 33449441 PMC8451754

[B22] Chapron S.-A. Kervran C. Da Rosa M. Fournet L. Shmulewitz D. Hasin D. (2023). Does food use disorder exist? Item response theory analyses of a food use disorder adapted from the DSM-5 substance use disorder criteria in a treatment seeking clinical sample. *Drug Alcohol Dependence* 251:110937. 10.1016/j.drugalcdep.2023.110937 37666092

[B23] Cherpitel C. J. Borges G. Ye Y. Bond J. Cremonte M. Moskalewicz J. (2010). Performance of a craving criterion in DSM alcohol use disorders. *J. Stud. Alcohol Drugs* 71 674–684. 10.15288/jsad.2010.71.674 20731972 PMC2930058

[B24] Chung T. Martin C. S. Maisto S. A. Cornelius J. R. Clark D. B. (2012). Greater prevalence of proposed DSM-5 nicotine use disorder compared to DSM-IV nicotine dependence in treated adolescents and young adults. *Addiction* 107 810–818. 10.1111/j.1360-0443.2011.03722.x 22092543 PMC3290741

[B25] Ciccarelli M. Cosenza M. Griffiths M. D. Nigro G. D’Olimpio F. (2019). Facilitated attention for gambling cues in adolescent problem gamblers: An experimental study. *J. Affect. Disord.* 252 39–46. 10.1016/j.jad.2019.04.012 30978623

[B26] Ciccarelli M. Cosenza M. Nigro G. D’Olimpio F. (2022). Does craving increase gambling severity? The role of attentional bias. *J. Affect. Disord.* 317 403–408. 10.1016/j.jad.2022.08.068 36029881

[B27] Collombat J. Chapron S.-A. Sarram S. Fatseas M. Serre F. Auriacombe M. (2024). L’anorexie mentale : Une addiction ? Application du modèle addictologique aux troubles du comportement alimentaire. Une revue narrative commentée. *L’Encéphale* 50 566–572. 10.1016/j.encep.2024.03.002 38755028

[B28] Cornil A. Lakritz C. Iceta S. Flaudias V. (2026). Craving in eating disorders: Mapping the concept through a systematic review. *Neurosci. Biobehav. Rev.* 181:106515. 10.1016/j.neubiorev.2025.106515 41371461

[B29] De-Sola J. Talledo H. Rubio G. De Fonseca F. R. (2017). Development of a mobile phone addiction craving scale and its validation in a Spanish adult population. *Front. Psychiatry* 8:90. 10.3389/fpsyt.2017.00090 28611692 PMC5447711

[B30] Del Mauro L. Vergallito A. Devoto F. Locatelli G. Hassan G. Romero Lauro L. J. (2025). Beyond the surface: Deep transcranial magnetic stimulation efficacy in reducing craving in addictive disorders: A systematic review and meta-analysis. *Biol. Psychiatry Cogn. Neurosci. Neuroimag.* 10 1005–1014. 10.1016/j.bpsc.2025.03.012 40204236

[B31] Demetrovics Z. Griffiths M. D. (2012). Behavioral addictions: Past, present and future. *J. Behav. Addict.* 1 1–2. 10.1556/JBA.1.2012.1.0 26166825

[B32] Dowling N. A. Merkouris S. S. Spence K. (2021). Ecological momentary assessment of the relationship between positive outcome expectancies and gambling behaviour. *J. Clin. Med.* 10:1709. 10.3390/jcm10081709 33921069 PMC8071390

[B33] el-Guebaly N. Mudry T. Zohar J. Tavares H. Potenza M. N. (2012). Compulsive features in behavioural addictions: The case of pathological gambling. *Addiction* 107 1726–1734. 10.1111/j.1360-0443.2011.03546.x 21985690 PMC3257403

[B34] Embretson S. E. Reise S. P. (2000). *Item Response Theory for Psychologists.* New York, NY: Psychology Press.

[B35] Enkema M. C. Hallgren K. A. Larimer M. E. (2020). Craving is impermanent and it matters: Investigating craving and cannabis use among young adults with problematic use interested in reducing use. *Drug Alcohol Depend.* 210:107957. 10.1016/j.drugalcdep.2020.107957 32200158 PMC7360486

[B36] Epskamp S. Rhemtulla M. Borsboom D. (2017). Generalized network psychometrics: Combining network and latent variable models. *Psychometrika* 82 904–927. 10.1007/s11336-017-9557-x 28290111

[B37] Fatseas M. Serre F. Alexandre J.-M. Debrabant R. Auriacombe M. Swendsen J. (2015). Craving and substance use among patients with alcohol, tobacco, cannabis or heroin addiction: A comparison of substance- and person-specific cues. *Addiction* 110 1035–1042. 10.1111/add.12882 25688760

[B38] Fauconnier M. Rousselet M. Brunault P. Thiabaud E. Lambert S. Rocher B. (2020). Food addiction among female patients seeking treatment for an eating disorder: Prevalence and associated factors. *Nutrients* 12:1897. 10.3390/nu12061897 32604734 PMC7353200

[B39] Ferreira G. M. Lee R. S. C. Piquet-Pessôa M. de Menezes G. B. Moreira-de-Oliveira M. E. Albertella L. (2021). Habitual versus affective motivations in obsessive-compulsive disorder and alcohol use disorder. *CNS Spectr.* 26 243–250. 10.1017/s1092852919001706 32041677

[B40] Field M. Kersbergen I. (2020). Are animal models of addiction useful? *Addiction* 115 6–12. 10.1111/add.14764 31353760

[B41] Fineberg N. A. Menchón J. M. Hall N. Dell’Osso B. Brand M. Potenza M. N. (2022). Advances in problematic usage of the internet research - A narrative review by experts from the European network for problematic usage of the internet. *Compr. Psychiatry* 118:152346. 10.1016/j.comppsych.2022.152346 36029549

[B42] Flayelle M. Fournier L. Billieux J. (2025). Renewing the assessment of behavioral addictions is necessary to avoid pathologizing nonproblematic behaviors: Commentary on Grubbs and Boness (2025). *J. Psychopathol. Clin. Sci.* 134 615–616. 10.1037/abn0001020 40658539

[B43] Fried E. I. (2015). Problematic assumptions have slowed down depression research: Why symptoms, not syndromes are the way forward. *Front. Psychol.* 6:309. 10.3389/fpsyg.2015.00309 25852621 PMC4369644

[B44] Garrison K. A. Sinha R. Potenza M. N. Gao S. Liang Q. Lacadie C. (2023). Transdiagnostic connectome-based prediction of craving. *Am. J. Psychiatry* 180 445–453. 10.1176/appi.ajp.21121207 36987598

[B45] Gauld C. Baillet E. Micoulaud-Franchi J.-A. Kervran C. Serre F. Auriacombe M. (2023). The centrality of craving in network analysis of five substance use disorders. *Drug Alcohol Depend.* 245:109828. 10.1016/j.drugalcdep.2023.109828 36868091

[B46] Gilder D. A. Gizer I. R. Lau P. Ehlers C. L. (2014). Item Response Theory analyses of DSM-IV and DSM-5 stimulant use disorder criteria in an American Indian community sample. *Drug Alcohol Depend.* 135 29–36. 10.1016/j.drugalcdep.2013.10.010 24200103 PMC3915779

[B47] Grant J. E. Potenza M. N. Weinstein A. Gorelick D. A. (2010). Introduction to behavioral addictions. *Am. J. Drug Alcohol Abuse* 36 233–241. 10.3109/00952990.2010.491884 20560821 PMC3164585

[B48] Grimm J. W. Fyall A. M. Osincup D. P. (2005). Incubation of sucrose craving: Effects of reduced training and sucrose pre-loading. *Physiol. Behav.* 84 73–79. 10.1016/j.physbeh.2004.10.011 15642609 PMC2880539

[B49] Hasin D. S. O’Brien C. P. Auriacombe M. Borges G. Bucholz K. Budney A. (2013). DSM-5 criteria for substance use disorders: Recommendations and rationale. *Am. J. Psychiatry* 170 834–851. 10.1176/appi.ajp.2013.12060782 23903334 PMC3767415

[B50] Hawker C. O. Merkouris S. S. Youssef G. J. Dowling N. A. (2021). Exploring the associations between gambling cravings, self-efficacy, and gambling episodes: An ecological momentary assessment study. *Addict. Behav.* 112:106574. 10.1016/j.addbeh.2020.106574 32759020

[B51] Heirene R. M. Shearer D. Roderique-Davies G. Mellalieu S. D. (2016). Addiction in extreme sports: An exploration of withdrawal states in rock climbers. *J. Behav. Addict.* 5 332–341. 10.1556/2006.5.2016.039 27348554 PMC5387785

[B52] Henry N. Pedersen M. Williams M. Donkin L. (2024). Quantifying the affective dynamics of pornography use and masturbation: An ecological momentary assessment study. *Res. Sq.* 10.21203/rs.3.rs-5094782/v1 [Preprint]. PMC12675693 41269492

[B53] Jones R. Bhattacharya J. (2012). Alpha activity in the insula accompanies the urge to neutralize in sub-clinical obsessive-compulsive participants. *J. Behav. Addict.* 1 96–105. 10.1556/jba.1.2012.005 26165459

[B54] Kennedy A. P. Epstein D. H. Jobes M. L. Agage D. Tyburski M. Phillips K. A. (2015). Continuous in-the-field measurement of heart rate: Correlates of drug use, craving, stress, and mood in polydrug users. *Drug Alcohol Depend.* 151 159–166. 10.1016/j.drugalcdep.2015.03.024 25920802 PMC4447529

[B55] Kervran C. Shmulewitz D. Serre F. Denis C. Roux P. Jauffret-Roustide M. (2021). Do DSM-5 substance use disorder criteria differ by user care settings? An item response theory analysis approach. *Addict. Behav.* 116:106797. 10.1016/j.addbeh.2020.106797 33450665

[B56] Kervran C. Shmulewitz D. Serre F. Stohl M. Denis C. Hasin D. (2020). Item response theory analyses of DSM-5 substance use disorder criteria in French outpatient addiction clinic participants. How much is craving special? *Drug Alcohol Depend.* 212:108036. 10.1016/j.drugalcdep.2020.108036 32464467

[B57] Kim H. S. Hodgins D. C. (2018). Component model of addiction treatment: A pragmatic transdiagnostic treatment model of behavioral and substance addictions. *Front. Psychiatry* 9:406. 10.3389/fpsyt.2018.00406 30233427 PMC6127248

[B58] Koob G. F. Volkow N. D. (2010). Neurocircuitry of addiction. *Neuropsychopharmacology* 35 217–238. 10.1038/npp.2009.110 19710631 PMC2805560

[B59] Koob G. F. Volkow N. D. (2016). Neurobiology of addiction: A neurocircuitry analysis. *Lancet Psychiatry* 3 760–773. 10.1016/S2215-0366(16)00104-8 27475769 PMC6135092

[B60] Kuss D. J. Griffiths M. D. (2012). Internet and gaming addiction: A systematic literature review of neuroimaging studies. *Brain Sci.* 2 347–374. 10.3390/brainsci2030347 24961198 PMC4061797

[B61] Mallorquí-Bagué N. Lozano-Madrid M. Testa G. Vintró-Alcaraz C. Sánchez I. Riesco N. (2020). Clinical and neurophysiological correlates of emotion and food craving regulation in patients with anorexia nervosa. *J. Clin. Med.* 9:960. 10.3390/jcm9040960 32244331 PMC7230937

[B62] Mallorqui-Bague N. Mestre-Bach G. Testa G. (2023). Craving in gambling disorder: A systematic review. *J. Behav. Addict.* 12 53–79. 10.1556/2006.2022.00080 36787136 PMC10260221

[B63] Meule A. Kübler A. (2012). Food cravings in food addiction: The distinct role of positive reinforcement. *Eat. Behav.* 13 252–255. 10.1016/j.eatbeh.2012.02.001 22664405

[B64] Miele C. Cabe J. Cabe N. Bertsch I. Brousse G. Pereira B. (2023). Measuring craving: A systematic review and mapping of assessment instruments. What about sexual craving? *Addiction* 118 2277–2314. 10.1111/add.16287 37493019

[B65] Moretta T. Buodo G. (2018). Autonomic stress reactivity and craving in individuals with problematic Internet use. *PLoS One* 13:e0190951. 10.1371/journal.pone.0190951 29338020 PMC5770068

[B66] Ort A. Wirz D. S. Fahr A. (2021). Is binge-watching addictive? Effects of motives for TV series use on the relationship between excessive media consumption and problematic viewing habits. *Addict. Behav. Rep.* 13:100325. 10.1016/j.abrep.2020.100325 33457488 PMC7797362

[B67] Panova T. Carbonell X. (2018). Is smartphone addiction really an addiction? *J. Behav. Addict.* 7 252–259. 10.1556/2006.7.2018.49 29895183 PMC6174603

[B68] Park S. Ha J. Ahn W. Kim L. (2023). Measurement of craving among gamers with internet gaming disorder using repeated presentations of game videos: A resting-state electroencephalography study. *BMC Public Health* 23:816. 10.1186/s12889-023-15750-4 37143023 PMC10158347

[B69] Petry N. Blanco C. Auriacombe M. Borges G. Bucholz K. Crowley T. (2013). An overview of and rationale for changes proposed for pathological gambling in DSM-5. *J. Gambling Stud.* 30 493–502. 10.1007/s10899-013-9370-0 23526033 PMC3722301

[B70] Petry N. M. Rehbein F. Gentile D. A. Lemmens J. S. Rumpf H. J. Mossle T. (2014). An international consensus for assessing internet gaming disorder using the new DSM-5 approach. *Addiction* 109 1399–1406. 10.1111/add.12457 24456155

[B71] Piquet-Pessoa M. Fontenelle L. F. (2016). Opioid antagonists in broadly defined behavioral addictions: A narrative review. *Expert Opin. Pharmacother.* 17 835–844. 10.1517/14656566.2016.1145660 26798982

[B72] Potenza M. N. (2008). The neurobiology of pathological gambling and drug addiction: An overview and new findings. *Philos. Trans. R. Soc. B: Biol. Sci.* 363 3181–3189. 10.1098/rstb.2008.0100 18640909 PMC2607329

[B73] Potenza M. N. (2009). Non-substance and substance addictions. *Addiction* 104 1016–1017. 10.1111/j.1360-0443.2009.02619.x 19466925 PMC2865686

[B74] Potenza M. N. (2014). Non-substance addictive behaviors in the context of DSM-5. *Addict. Behav.* 39 1–2. 10.1016/j.addbeh.2013.09.004 24119712 PMC3858502

[B75] Quintero M. J. Navas J. F. Perales J. C. (2020). The associative underpinnings of negative urgency and its role in problematic gambling behavior. *Addict. Behav.* 111:106533. 10.1016/j.addbeh.2020.106533 32771795

[B76] Robinson T. E. Berridge K. C. (2008). Review. The incentive sensitization theory of addiction: Some current issues. *Philos. Trans. R. Soc. Lond. B Biol. Sci.* 363 3137–3146. 10.1098/rstb.2008.0093 18640920 PMC2607325

[B77] Roefs A. Boh B. Spanakis G. Nederkoorn C. Lemmens L. H. J. M. Jansen A. (2019). Food craving in daily life: Comparison of overweight and normal-weight participants with ecological momentary assessment. *J. Hum. Nutrit. Diet.* 32 765–774. 10.1111/jhn.12693 31430000 PMC6899849

[B78] Sahin A. Anil D. (2017). The effects of test length and sample size on item parameters in item response theory. *Educ. Sci. Theory Pract.* 17 321–335. 10.12738/estp.2017.1.0270

[B79] Sayette M. A. (2016). The role of craving in substance use disorders: Theoretical and methodological issues. *Ann. Rev. Clin. Psychol.* 12 407–433. 10.1146/annurev-clinpsy-021815-093351 26565121

[B80] Sayette M. A. Shiffman S. Tiffany S. T. Niaura R. S. Martin C. S. Shadel W. G. (2000). The measurement of drug craving. *Addiction* 95(Suppl. 2), S189–S210. 10.1080/09652140050111762 11002914 PMC2683662

[B81] Schlechter P. Wilkinson O. Knausenberger J. Wanninger K. Kamp S. Morina N. (2021). Depressive and anxiety symptoms in refugees: Insights from classical test theory, item response theory and network analysis. *Clin. Psychol. Psychotherapy* 28 169–181. 10.1002/cpp.2499 32808440

[B82] Serre F. Fatseas M. Debrabant R. Alexandre J.-M. Auriacombe M. Swendsen J. (2012). Ecological momentary assessment in alcohol, tobacco, cannabis and opiate dependence: A comparison of feasibility and validity. *Drug Alcohol Depend.* 126 118–123. 10.1016/j.drugalcdep.2012.04.025 22647899

[B83] Serre F. Fatseas M. Denis C. Swendsen J. Auriacombe M. (2018). Predictors of craving and substance use among patients with alcohol, tobacco, cannabis or opiate addictions: Commonalities and specificities across substances. *Addict. Behav.* 83 123–129. 10.1016/j.addbeh.2018.01.041 29428330

[B84] Serre F. Fatseas M. Swendsen J. Auriacombe M. (2015). Ecological momentary assessment in the investigation of craving and substance use in daily life: A systematic review. *Drug Alcohol Depend.* 148C 1–20. 10.1016/j.drugalcdep.2014.12.024 25637078

[B85] Serre F. Gauld C. Lambert L. Baillet E. Beltran V. Daulouede J. P. (2025). Predictors of substance use during treatment for addiction: A network analysis of ecological momentary assessment data. *Addiction* 120 48–58. 10.1111/add.16658 39210697 PMC11638523

[B86] Shaffer H. LaPlante D. Kidman R. Donato A. Stanton M. (2004). Toward a syndrome model of addiction: Multiple expressions, common etiology. *Harvard Rev. Psychiatry* 12 367–374. 10.1080/10673220490905705 15764471

[B87] Shin Y.-B. Kim J.-J. Kim M.-K. Kyeong S. Jung Y. H. Eom H. (2018). Development of an effective virtual environment in eliciting craving in adolescents and young adults with internet gaming disorder. *PLoS One* 13:e0195677. 10.1371/journal.pone.0195677 29672530 PMC5908156

[B88] Shmulewitz D. Levitin M. D. Eliashar R. Mikulincer M. Lev-Ran S. (2025). Network analysis of Alcohol, Smoking and Substance Involvement Screening Test (ASSIST) 3.1 items for non-medical use of alcohol, tobacco, cannabis, prescription sedatives, prescription stimulants, and prescription opioids. *Front. Psychiatry* 16:1541628. 10.3389/fpsyt.2025.1541628 40454390 PMC12122760

[B89] Shmulewitz D. Stohl M. Greenstein E. Roncone S. Walsh C. Aharonovich E. (2023). Validity of the DSM-5 craving criterion for alcohol, tobacco, cannabis, cocaine, heroin, and non-prescription use of prescription painkillers (opioids). *Psychol. Med.* 53 1955–1969. 10.1017/S0033291721003652 35506791 PMC9096712

[B90] Starcevic V. (2016). Tolerance and withdrawal symptoms may not be helpful to enhance understanding of behavioural addictions. *Addiction* 111 1307–1308. 10.1111/add.13381 27095413

[B91] Starcevic V. Billieux J. (2017). Does the construct of Internet addiction reflect a single entity or a spectrum of disorders? *Clin. Neuropsychiatry* 14 5–10.

[B92] Stone A. A. Shiffman S. (1994). Ecological Momentary Assessment (EMA) in behavioral medicine. *Ann. Behav. Med.* 16 199–202. 10.1093/abm/16.3.199

[B93] Tomova L. Wang K. L. Thompson T. Matthews G. A. Takahashi A. Tye K. M. (2020). Acute social isolation evokes midbrain craving responses similar to hunger. *Nat. Neurosci.* 23 1597–1605. 10.1038/s41593-020-00742-z 33230328 PMC8580014

[B94] Vafaie N. Kober H. (2022). Association of drug cues and craving with drug use and relapse: A systematic review and meta-analysis. *JAMA Psychiatry* 79:641. 10.1001/jamapsychiatry.2022.1240 35648415 PMC9161117

[B95] Volkow N. D. Blanco C. (2023). Substance use disorders: A comprehensive update of classification, epidemiology, neurobiology, clinical aspects, treatment and prevention. *World Psychiatry* 22 203–229. 10.1002/wps.21073 37159360 PMC10168177

[B96] Volkow N. D. Koob G. F. McLellan A. T. (2016). Neurobiologic advances from the brain disease model of addiction. *N. Engl. J. Med.* 374 363–371. 10.1056/NEJMra1511480 26816013 PMC6135257

[B97] Westerberg T. Hakansson A. Berglund K. (2025). The role of craving in gambling behavior: Examining its relevance and links to psychological distress, personality, and demographics. *BMC Psychol.* 14:74. 10.1186/s40359-025-03816-4 41388562 PMC12810000

[B98] World Health Organization (2018). *ICD-11: International Classification of Diseases (11th revision).* Geneva: WHO.

[B99] Young K. S. (1998). Internet addiction: The emergence of a new clinical disorder. *CyberPsychol. Behav.* 1 237–244. 10.1089/cpb.1998.1.237

[B100] Zhang J. Chen S. Jiang Q. Dong H. Zhao Z. Du X. (2021). Disturbed craving regulation to gaming cues in internet gaming disorder: Implications for uncontrolled gaming behaviors. *J. Psychiatr. Res.* 140 250–259. 10.1016/j.jpsychires.2021.05.051 34119910

[B101] Zhou Y. Zhou Y. Zhou J. Shen M. Zhang M. (2022). Attentional biases and daily game craving dynamics: An ecological momentary assessment study. *J. Behav. Addict.* 11 1044–1054. 10.1556/2006.2022.00085 36427198 PMC9881657

